# Intestinal Ultrasound-Guided Precision Medicine in Inflammatory Bowel Diseases: A Narrative Review

**DOI:** 10.3390/jpm16070339

**Published:** 2026-06-23

**Authors:** Cicerone Clelia, Fabrizio Fanizzi, Arianna Dal Buono, Ilaria Faggiani, Ferdinando D’Amico, Alessandra Zilli, Tommaso Lorenzo Parigi, Virginia Solitano, Federica Furfaro, Sara Massironi, Alessandro Armuzzi, Silvio Danese, Mariangela Allocca

**Affiliations:** 1 Department of Gastroenterology and Endoscopy, IRCCS Hospital San Raffaele, 20132 Milan, Italy; 2 Faculty of Medicine and Surgery, Vita e Salute San Raffaele University, 20132 Milan, Italy; 3 IBD Center, IRCCS Humanitas Research Hospital, Via Manzoni 56, 20089 Rozzano, Italy; 4 Department of Biomedical Sciences, Humanitas University, Pieve Emanuele, 20072 Milan, Italy; 5 Gastroenterology Unit, Istituti Ospedalieri Bergamaschi, Zingonia, 24046 Bergamo, Italy

**Keywords:** IBD, inflammatory bowel disease, IUS, intestinal ultrasound, precision medicine, personalized medicine

## Abstract

Inflammatory bowel diseases (IBD), including Crohn’s disease and ulcerative colitis, are characterized by marked heterogeneity, challenging disease monitoring and individualized treatment. Despite advances in treat-to-target strategies, unmet needs persist, particularly in assessing transmural healing and optimizing therapeutic decisions. This narrative review evaluates the role of intestinal ultrasound (IUS) as a key tool for precision medicine in IBD. IUS is a non-invasive, repeatable, and cost-effective imaging modality with diagnostic accuracy comparable to endoscopy and magnetic resonance enterography, with reported sensitivities and specificities frequently exceeding 80–90% for detecting active disease. It enables real-time assessment of transmural inflammation and complications, while parameters such as bowel wall thickness and Doppler vascularity support prognostic stratification. Early reductions in bowel wall thickness (≥25–30%) have been associated with improved treatment response, allowing identification of responders within weeks of therapy initiation. IUS informs therapeutic decision-making, including initiation, optimization, and de-escalation of advanced therapies, and may reduce reliance on invasive procedures. Integration into routine care has been associated with improved disease control and cost-effectiveness. Standardization of protocols, operator training, and prospective validation are required to establish IUS as a cornerstone of precision medicine in IBD.

## 1. Introduction

Inflammatory bowel diseases (IBD), encompassing Crohn’s disease (CD) and ulcerative colitis (UC), are chronic, immune-mediated disorders characterized by substantial heterogeneity in clinical presentation, disease behavior, and therapeutic response [1,2]. This variability reflects interactions among genetic predisposition, environmental triggers, microbiome alterations, and immune dysregulation, leading to diverse disease trajectories and treatment outcomes [3]. As a result, uniform therapeutic strategies are often insufficient, and there is a growing need for individualized dynamic approaches of disease monitoring and management [4,5,6]. Despite advances in biologic and small molecule therapies, several unmet needs persist in routine IBD care [7,8]. Clinical indices frequently underestimate inflammatory burden [9], while endoscopy, despite being the gold standard for mucosal assessment, is invasive, costly, and impractical for frequent monitoring [10,11]. Biomarkers such as C-reactive protein (CRP) and fecal calprotectin (FC) provide useful but incomplete information, particularly regarding transmural inflammation and structural complications [12]. These limitations hinder the implementation of personalized, tight control strategies to optimize long-term outcomes [13]. The treat to target (T2T) paradigm has reshaped IBD management by emphasizing predefined therapeutic goals, including symptomatic remission, biomarker normalization, and mucosal healing (MH) [10]. However, in CD, where inflammation involves the entire bowel wall, MH alone may not fully reflect true disease resolution [14]. Cross-sectional imaging studies have demonstrated that transmural inflammation can remain despite endoscopic remission, contributing to stricturing, penetrating complications, and long-term bowel damage [15].

Transmural healing (TH) is increasingly recognized as a meaningful therapeutic goal in CD, associated with improved long-term outcomes compared to MH alone [14,16,17]. Intestinal ultrasound (IUS) reliably assesses TH and treatment response, offering standardized criteria for clinical practice and research [15,16]. Achieving TH has been associated with lower rates of hospitalization, surgery, corticosteroid exposure, and disease progression, suggesting that it may represent a superior long-term target compared with MH alone [16]. Within this evolving therapeutic landscape, precision medicine has become central to IBD management [18,19]. Precision strategies aim to tailor therapeutic decisions by integrating clinical phenotype, molecular and multiomic signatures, pharmacokinetic and pharmacodynamic profiles, and imaging findings [20,21]. Recent multiomic and machine-learning studies have shown that patient-specific biological signatures can meaningfully predict treatment response, risk of complications, and likelihood of achieving deep or transmural remission [22,23]. Among the available modalities, IUS has gained prominence due to its non-invasiveness, absence of radiation, real-time availability, and suitability for repeated assessments [24,25]. IUS enables point-of-care evaluation of bowel wall thickness, vascularity, mesenteric changes, and complications, making it an ideal tool for tight-control and T2T strategies [26,27]. The STRIDE-II initiative has further emphasized objective targets, including TH [10,28]. Within this framework, IUS has emerged as a pivotal tool capable of supporting both short- and long-term treatment goals.

This narrative review aims to define the principles and clinical implications of precision medicine in IBD, outline the rationale for imaging-guided management and highlight the advantages and emerging role of IUS as a cornerstone of precision, real-time disease monitoring in IBD.

## 2. Materials and Methods

A comprehensive literature search was conducted to identify studies evaluating the role of IUS in IBD within the framework of precision medicine. Electronic databases, including PubMed/MEDLINE, Embase, and Scopus, were searched from inception to March 2026. The search strategy combined terms related to IBD, IUS, and precision medicine, including “inflammatory bowel disease”, “Crohn’s disease”, “ulcerative colitis”, “intestinal ultrasound”, “bowel ultrasound”, “point-of-care ultrasound”, “precision medicine”, “personalized medicine”, “treat-to-target”, “disease monitoring”, and “transmural healing”. Only articles published in English were considered. Original studies, systematic reviews, meta-analyses, consensus statements, and clinical practice guidelines were eligible for inclusion. Publications not relevant to the topic of IUS in precision medicine or lacking sufficient methodological detail were excluded. Titles and abstracts were screened independently by two reviewers, followed by full-text assessment of potentially relevant articles. Reference lists of selected articles and relevant reviews were manually searched to identify additional pertinent studies. Given the narrative nature of the review, no formal quality assessment or evidence grading system was applied. Studies were included based on their scientific relevance and contribution to the understanding of IUS as a tool for disease characterization, monitoring, prediction of therapeutic response, and implementation of precision medicine strategies in IBD.

## 3. Results

### 3.1. Intestinal Ultrasound in IBD: Technical Background

IUS has evolved into a highly reliable, non-invasive imaging modality for the assessment of IBD [29]. Modern high-frequency transducers (5–17 MHz) allow detailed visualization of bowel wall layers, mesenteric structures, and vascular patterns [29,30]. The technique is well tolerated, requires no bowel preparation, and can be performed repeatedly at the point of care, making it particularly suitable for tight-control and T2T strategies [29,31]. Advances in Doppler technology, contrast-enhanced ultrasound (CEUS), and elastography have further improved the sensitivity for detecting active inflammation and structural complications [32,33]. IUS provides real-time assessment of several validated parameters that correlate with disease activity and transmural inflammation [29,34,35,36]:

- **Bowel Wall Thickness (BWT).** BWT is the most widely used and reproducible IUS parameter. Increased thickness reflects active inflammation and correlates with endoscopic and radiologic disease activity. Thresholds of >3 mm both in the terminal ileum and in all colonic segments are generally considered abnormal in adults [37,38].
- **Color Doppler Signal (CDS).** Color Doppler assesses mural hyperemia, a hallmark of active inflammation. Semi-quantitative grading systems (e.g., Limberg score and modified Limberg score) are increasingly used in clinical practice and research. Doppler signal reduction is a sensitive marker of therapeutic response [39,40].
- **Mesenteric Changes.** IUS can detect mesenteric fat hypertrophy, increased echogenicity, and enlarged lymph nodes, features associated with CD activity and complications. These findings complement mural parameters and improve diagnostic accuracy [41,42].
- **Bowel Wall Stratification (BWS).** Loss of stratification suggests severe inflammation, whereas preservation of layers may help differentiate inflammatory from fibrotic strictures [42,43].

### 3.2. Comparative Accuracy of Intestinal Ultrasound, Magnetic Resonance Enterography, and Endoscopy in Assessment of IBD Activity

IUS demonstrates strong correlation with both endoscopic indices of inflammation and cross-sectional imaging modalities such as magnetic resonance enterography (MRE) [32,44]. Several studies have shown that key IUS parameters, including BWT, Doppler vascularity, loss of stratification, and mesenteric inflammatory changes, closely mirror endoscopic severity in both CD and UC [45,46]. Although IUS cannot directly visualize mucosal ulcerations, its ability to detect transmural inflammation provides complementary and, in some cases, incremental information beyond mucosal assessment alone, particularly in CD. Multiple studies have demonstrated high concordance between IUS and MRE for detecting active inflammation, strictures, fistulas, and abscesses [47]. Across cross-sectional imaging modalities, both IUS and MRE consistently show moderate to high diagnostic accuracy when compared with endoscopy or composite reference standards, with reported sensitivity and specificity frequently exceeding 60–90% depending on disease location and severity [26]. Ripollés et al. demonstrated excellent agreement between BWT, Doppler signal, and MRE inflammation markers [48]. The METRIC trial demonstrated that IUS has sensitivity comparable to MRE for the detection of active small bowel CD [49], supporting its role as a first-line, non-invasive alternative for disease assessment in clinical practice. Crucially, reduction in BWT, vascularity, and inflammatory mesenteric changes on IUS has been associated with clinical response [50], biochemical improvement, and radiologic healing on MRE, indicating that IUS can reliably track treatment response over time [51] and capture TH rather than mucosal changes alone [39,52]. Sagami et al. performed a systematic review and meta-analysis demonstrating that IUS has high sensitivity and specificity for detecting active CD, with BWT representing the most reliable parameter, further reinforcing its robustness as a diagnostic and monitoring tool [37]. Furthermore, IUS correlates not only with radiologic indices but also with endoscopic severity. A strong correlation between BWT and Simple Endoscopic Score for Crohn’s Disease (SES-CD), particularly for ileal disease, was repeatedly reported in prospective studies [53,54], with Bots et al. confirming that IUS parameters (BWT, vascularity, and mesenteric fat changes) predict endoscopic ulceration [55]. Overall, available evidence demonstrates a strong concordance between IUS, MRE and endoscopy for the assessment of inflammatory activity in IBD. While endoscopy remains the reference standard for mucosal evaluation, IUS and MRE provide complementary transmural information, with IUS offering the additional advantages of point-of-care availability, repeatability, and low patient burden (Table 1).

### 3.3. Intestinal Ultrasound Scores for Assessing Activity in Crohn’s Disease and Ulcerative Colitis

IUS has evolved into a structured, quantifiable tool for assessing IBD, supported by several validated scoring systems [41,56]. In CD, most scores focus on transmural inflammation, integrating BWT, Doppler vascularity, wall stratification, and mesenteric inflammatory changes, which have been identified through international expert consensus as the core sonographic parameters of disease activity [41]. The most widely used in CD include the international bowel ultrasound group-segmental activity score (IBUS-SAS), a composite score derived from a multivariable model (4 × BWT + 15 × inflammatory fat + 7 × Doppler signal + 4 × stratification) with excellent interobserver reliability (ICC ≈ 0.97) [41], the bowel ultrasound score (BUSS) [57], and the Simple Ultrasound Activity Score (SUS-CD) [58], all designed to provide reproducible segmental assessment of disease activity [58,59].

Comparative studies have demonstrated that all major IUS scores significantly correlate with endoscopic activity, supporting its potential role as a reference standard in expert centers [56,59]. In UC, disease-specific scores have also been developed, including the Milan Ultrasound Criteria (MUC-UC) [60,61] and the IBUS-SAS adapted for UC [62,63]. Elastography-based scores represent an emerging frontier for assessing bowel wall stiffness and fibrosis, particularly in CD [32,44,64]. However, current evidence remains heterogeneous and largely exploratory, with a lack of standardized acquisition protocols, validated cut-offs, and large prospective studies, limiting their routine clinical implementation (Table 2).

### 3.4. Patient Stratification and Prognostic Value

The clinical relevance of IUS extends beyond its role as a real-time tool for assessing inflammatory activity, disease extent, and response to therapy. In fact, IUS enables the identification of patients at increased risk of relapse, as well as those at risk of developing acute severe ulcerative colitis (ASUC), by detecting persistent transmural and extramural inflammation. This underpins the well-established prognostic value of IUS [36,65]. Imaging features associated with both active disease and an unfavorable prognosis include marked BWT, loss of BWS, mesenteric fat hypertrophy, and enlarged mesenteric lymph nodes, all of which are indicative of severe inflammatory burden [36]. This prognostic information is highly relevant in clinical practice, as it contributes to patient risk stratification by identifying individuals at higher or lower risk of persistent disease activity, clinical deterioration, and adverse outcomes, including the need for colectomy. This allows a more tailored monitoring strategy, helping clinicians determine the intensity and timing of follow-up assessments, including biomarker evaluation, subsequent IUS or MRI, and endoscopic reassessment [36].

In UC, the prognostic role of IUS may be particularly important when linked to hard clinical outcomes such as surgery. A systematic review by Josefsen et al. including 18 prospective studies evaluated the role of IUS as a predictor of treatment response, remission, relapse, and adverse outcomes in adult patients with UC in outpatient (11 studies) and hospitalized settings (7 studies, including patients with ASUC treated with intravenous corticosteroids [IVCS]). Among these latter patients, changes in BWT within 48–72 h after treatment initiation proved to be the strongest indicator of disease trajectory and treatment response [66]. In particular, a BWT ≥4 mm, an absolute reduction of ≤1 mm, or a relative reduction of ≤20% at 48 h showed good discriminative performance for identifying patients who would require rescue therapy. In addition, persistently elevated BWT on day 6 was associated with a significantly increased risk of colectomy, whereas achievement of a BWT < 3 mm at 48 h was associated with the absence of surgeries [66]. This predictive role of BWT for clinical outcomes is also maintained in the outpatient setting, where BWT values ≤ 3.6 mm at two weeks and <3.0 mm at six weeks after treatment initiation were associated with early endoscopic remission. Conversely, persistent BWT > 3.5 mm or minimal reduction in wall thickness (<20% or <1 mm) indicated a low probability of long-term remission. Beyond single parameters, composite ultrasound indices incorporating vascularity further improved predictive performance. In particular, the MUC score demonstrated strong prognostic value for long-term outcomes; a score ≤ 4.3 or a reduction of at least two points at 12 weeks predicted sustained remission, whereas values ≥ 7.7 identified patients at high risk of treatment failure or colectomy. These findings support the incorporation of IUS into early treatment-response monitoring algorithms in UC [66].

Comparable prognostic applications of IUS have also been described in CD [67]. Zorzi et al. showed that TH, assessed by ultrasound, is a valuable marker in CD and may be linked to better clinical outcomes, supporting its use in routine disease monitoring [68]. Notably, in CD, the value of IUS lies not only in the assessment of disease activity but also in its ability to capture the stricturing and penetrating phenotypes that characterize the disease, with accuracy comparable to MRE and CT for identifying strictures, fistulae, and abscesses [69]. Advanced IUS techniques, such as contrast-enhanced ultrasound (CEUS), may further improve accuracy in identification of complication. Indeed, through real-time evaluation of bowel wall perfusion and transmural inflammatory activity following intravenous contrast administration, CEUS enhances the ability of IUS to detect penetrating disease and to differentiate abscesses from phlegmons, showing excellent agreement with other imaging modalities (κ = 0.972) [70].

On the other hand, a prospective, cross-sectional study including patients with stenotic CD who underwent IUS prior to a small bowel segment resection, showed how loss of wall layer stratification (WLS) (OR 7.86, *p* = 0.029) and higher CEUS perfusion parameters were associated with inflammatory strictures. In contrast, chronic strictures had lower BWT (5.74 vs. 7.46 mm, *p* = 0.002), as well as lower CDS (OR 0.14, *p* = 0.03) and less frequent loss of WLS (OR 0.14, *p* = 0.027). Based on these parameters, the Stricture Score Amsterdam was developed, and, although it has not yet been externally validated, it showed good diagnostic accuracy for identifying inflammatory (AUC 0.88, *p* = 0.002) and chronic strictures (AUC 0.90, *p* < 0.0001), with good inter-observer agreement (ICC 0.73, *p* < 0.0001) [71].

Another helpful technique in the assessment of fibrosis may be represented by elastography, which measures bowel wall stiffness, with higher values suggesting fibrosis. While elastography is promising for phenotyping strictures, current consensus highlights methodological limitations and lack of standardization, so color Doppler and CEUS remain the primary adjunctive modalities for improving the ability of IUS to assess transmural inflammatory activity and to differentiate between inflammatory and fibrotic strictures, guiding decisions between medical and surgical management [72].

### 3.5. IUS-Guided Therapeutic Decision-Making

#### 3.5.1. IUS Integration in Treat-to-Target Strategies

IUS is a valuable tool for guiding treat-to-target strategies in IBD, enabling timely and individualized therapeutic decisions based on combined imaging and clinical data, without relying solely on endoscopy. It detects subclinical disease activity and supports real-world management. In routine practice, IUS may inform escalation, de-escalation, or switching of therapy, particularly in asymptomatic patients or in cases of discordant clinical and biochemical findings [27,73]. Supporting this, a retrospective cohort study by Jud et al. at a Swiss tertiary center evaluated 103 adult IBD patients (66% CD, 34% UC). IUS assessment of BWT in the terminal ileum and left colon significantly influenced treatment decisions (*p* = 0.014 and *p* = 0.042, respectively). In multivariable logistic regression analysis, both the presence of diarrhea (*p* = 0.013) and maximal BWT (*p* = 0.020) remained independent predictors of a change in therapy [74]. These findings are supported by additional real-world and prospective studies demonstrating the feasibility of integrating IUS into routine clinical decision-making. A retrospective study of 447 outpatient IUS examinations at a tertiary IBD center, showed that abnormal IUS findings prompted treatment changes more frequently in symptomatic patients than in asymptomatic ones (67% vs. 35%; *p* < 0.001). When decisions were guided by IUS, clinicians ordered fewer additional investigations, stool tests (9% vs. 51%), imaging (4% vs. 22%), and endoscopy (9% vs. 39%; all *p* < 0.0001). IUS findings also correlated strongly with FC (*p* < 0.0001), endoscopy (*p* = 0.002), and MRI (*p* = 0.047), confirming its role in optimizing patient management while minimizing unnecessary interventions [25]. Prospective evidence also supports the feasibility of point-of-care (POC) IUS in clinical practice. When IUS findings and biomarkers were concordant, IUS-based decision-making ranged from 66.3% to 76.4%, leading to management changes in approximately 45% of patients. Importantly, even in cases with discordant IUS and biomarker results, the initial management decision based on IUS remained unchanged in nearly 80% of patients [75]. Beyond its impact on therapeutic decision-making in mixed IBD cohorts, accumulating evidence supports the prognostic value of IUS across individual disease phenotypes, including UC [76]. This concept was demonstrated in a prospective multicenter cohort study by Madsen et al. including 193 newly diagnosed patients with UC. At diagnosis, IUS findings correlated closely with symptomatic, biochemical, and endoscopic markers of inflammation and independently predicted the need for colectomy within the first 3 months, with BWT > 6 mm emerging as the optimal threshold for predicting early surgery (OR 38; 95% CI 8–270; *p* < 0.0001). Importantly, IUS also proved useful for monitoring early therapeutic response. After 3 months of treatment, 59% of patients achieved transmural remission, defined as BWT ≤ 3 mm in the absence of Doppler vascular signal. Patients achieving this ultrasonographic target showed higher rates of steroid-free clinical remission, lower steroid exposure during follow-up, and a greater likelihood of maintaining remission at 12 months [77]. Ongoing studies, such as the phase 4 randomized VECTORS trial, are further supporting the feasibility of incorporating IUS into a treat-to-target strategy [78].

#### 3.5.2. Early Sonographic Predictors of Response

Recent real-world studies and prospective cohorts highlight that early reductions in BWT and normalization of Doppler signals within 2–6 weeks after therapy initiation correlate strongly with endoscopic response and remission, allowing identification of responders and non-responders, minimizing exposure to ineffective agents [79].

Evidence from interventional trials supports the ability of IUS to detect therapeutic response very early during biologic therapy. In the STARDUST trial, a randomized controlled study comparing treat-to-target versus standard-of-care strategies in patients treated with ustekinumab, IUS was used to evaluate transmural inflammation over time. Significant reductions in BWT were already observed by week 4, with an IUS response (≥25% BWT reduction) in 46.3% of patients and transmural remission in 24.1% by week 48 [45]. Consistent with these findings, a multicenter prospective Italian study involving 188 CD patients treated with biologics demonstrated that IUS can reliably track improvements in bowel inflammation and the achievement of TH over time. BWT significantly improved as early as 3 months (*p* < 0.0001), and TH at 12 months ranged from 20% with ustekinumab to 37% with infliximab (number needed to treat 3.6). Moreover, colonic disease predicted higher rates of early healing, whereas greater baseline BWT was associated with a lower probability of achieving transmural remission. These results reinforce the role of IUS as a practical tool for longitudinal monitoring of biologic-induced disease modification [67]. Similarly, in a retrospective cohort of CD, patients who experienced treatment failure had significantly greater BWT (5 mm vs. 2.8 mm; *p* < 0.0001), and a thickness ≥4 mm independently predicted loss of response (OR 2.9; 95% CI 1.49–5.55; *p* = 0.002). These findings suggest that maintaining BWT below this threshold may serve as a practical therapeutic goal during biologic treatment [80]. Similar findings have been reported for anti-TNF therapy for CD patients. De Voogd et al. evaluated IUS and CEUS for predicting early endoscopic response in 40 patients. Reductions in BWT within 4–8 weeks predicted endoscopic response and remission, with a T2 BWT ≤ 3.2 mm most accurately identifying remission. CEUS added limited predictive value, while absence of color Doppler signal modestly improved prediction [81]. In another study, Chen et al. combined conventional IUS with shear wave elastography (SWE) to assess early response to anti-TNF therapy in patients with CD. Among 30 patients, reductions in BWT, Doppler signals evaluated with Limberg score, and SWE values were detectable as early as week 2 and strongly associated with response at week 14. Baseline BWT and SWE values were higher in non-responders, suggesting predictive utility for patient stratification [82].

Other investigations highlight the prognostic value of IUS later in the first phase of disease course. In a prospective population-based cohort of 201 newly diagnosed CD patients, transmural remission at 3 months, achieved in 38% of patients, was strongly associated with steroid-free clinical remission and a significantly lower risk of treatment escalation at 12 months (26% vs. 53%; *p* = 0.003). Notably, IUS findings at diagnosis also demonstrated strong predictive capacity: the IBUS-SAS score in the terminal ileum predicted the need for ileocecal resection within the first year with excellent accuracy (AUC 0.92). These data support the concept that early transmural healing may represent a meaningful therapeutic target and prognostic marker [83].

Extending these observations to UC, similar prospective studies have highlighted the value of early IUS assessment for predicting treatment response. In the TRUST&UC study, Maaser et al. evaluated patients with UC experiencing clinical relapse across 42 German IBD-specialized centers. At baseline, 88.5% of patients showed increased BWT in the sigmoid or descending colon. BWT decreased significantly within the first 2 weeks of therapy (sigmoid: from 89.3% to 38.6%; descending: from 83.0% to 42.9%; *p* < 0.001) and remained low at weeks 6 and 12. Notably, normalization of BWT strongly correlated with clinical response at week 12, with 90.5% of patients demonstrating symptomatic improvement (*p* < 0.001), supporting IUS as a reliable early surrogate marker of therapeutic efficacy in UC [39]. Complementing these findings, De Voogd et al. prospectively assessed 51 patients with UC (Mayo endoscopic score ≥2) at baseline, weeks 2 and 6, and at a second endoscopy between weeks 8 and 26. Reductions in BWT and the presence of color Doppler signal at week 6 were strong predictors of endoscopic improvement and remission, with BWT ≤ 3.0 mm markedly increasing the likelihood of endoscopic remission (OR 25.13). Drug-specific kinetics were observed; infliximab and tofacitinib induced significant BWT reductions by week 2, whereas vedolizumab showed delayed effects at week 6 [84]. A parallel prospective study evaluating early sonographic response after initiation of biologic or thiopurine therapy in IBD patients confirmed that BWT reduction at 6 and 14 weeks predicted long-term treatment success, outperforming conventional clinical indices and CRP, while fecal calprotectin retained some predictive value [85]. Importantly, given the limitations of transabdominal ultrasound in rectal assessment, transperineal ultrasound (TPUS), performed with the probe positioned directly over the perineal region, may further improve the evaluation of very early treatment response in UC, particularly in cases with rectal involvement. In a prospective study, early changes as soon as week 1, specifically a reduction in rectal BWT, were independently associated with clinical remission at week 8, outperforming baseline IUS parameters and conventional biomarkers. Notably, this predictive value persisted even in patients without initial clinical improvement, highlighting the potential of TPUS as an ultra-early, non-invasive tool to anticipate treatment response [86].

#### 3.5.3. Integration with Therapeutic Drug Monitoring

Therapeutic drug monitoring (TDM) integrated with IUS may enable correlation between biologic drug exposure and transmural inflammatory activity, supporting treatment optimization in IBD. A prospective study by Vaughan et al. investigated the relationship between maintenance infliximab trough levels (ITLs) and IUS-assessed transmural healing in 103 IBD patients (79 CD and 24 UC). In CD, patients achieving sonographic healing (BWT ≤ 3 mm, no increased Doppler signal) had higher median ITLs than those with inflammation (4.8 vs. 3.1 μg/mL; *p* = 0.049), and Doppler hyperemia was independently associated with lower ITLs (2.1 vs. 4.2 μg/mL; *p* = 0.003). In UC, lower ITLs were linked to Doppler hyperemia (*p* = 0.04), but no significant association was observed with overall sonographic healing. These results suggest that lower infliximab levels correlate with persistent sonographic inflammation, supporting the potential value of optimizing ITLs to achieve transmural healing [87]. Accordingly with these findings, a prospective study by Ungar et al. examined the association between adalimumab trough levels and IUS-measured BWT in 44 CD patients (50 paired measurements). Patients with trough levels <3 μg/mL had significantly higher BWT in both the terminal ileum (*p* = 0.04) and colon (*p* = 0.02). Additionally, therapy retention over one year was higher in patients with terminal ileum thickness <4 mm (*p* = 0.03). These findings suggest that lower adalimumab levels correlate with increased transmural inflammation, supporting IUS as a noninvasive tool to guide biologic therapy in CD [88].

### 3.6. IUS Role in Preoperative and Postoperative Monitoring

IUS enables timely risk stratification, guides therapeutic decision-making, and supports treat-to-target strategies in newly diagnosed patients as well as in the postoperative setting, given its value for assessing recurrence in operated CD patients. In a systematic review by Pal et al. including 20 studies, BWT emerged as a robust predictor of endoscopic recurrence. Thresholds of ≥5 mm at the neo-terminal ileum demonstrated sensitivities ranging from 81% to 94% and specificities from 86% to 100%. Lower cut-offs at the ileocolonic anastomosis (≥3–3.5 mm) were also associated with increased risk, although data were primarily from single-center cohorts. Assessing both the neo-terminal ileum and the anastomosis (dual-site evaluation) improved diagnostic performance. Additional sonographic features, such as Doppler-detected hyperemia and mesenteric lymphadenopathy, further increased predictive accuracy. Notably, combining a BWT ≥ 3 mm with FC ≥ 50 μg/g achieved very high specificity (approximately 93–100%) and a negative predictive value of nearly 95% when both parameters were negative. Composite scores based on CEUS reached approximately 98% diagnostic accuracy in prospective cohorts. Similarly, small intestine contrast ultrasound (SICUS) showed strong early diagnostic performance, with area under the receiver operating characteristic (AUROC) values up to 0.95 when combining anastomotic BWT ≥ 3–3.5 mm with lesion length and sensitivities of 82–94% and specificities > 90% reported even within 7 days post-resection. Overall, IUS demonstrated moderate agreement with endoscopy (κ approximately 0.5–0.8) and appeared to have stronger prognostic value when performed within the first 12 months post-surgery. These findings support the integration of IUS into postoperative surveillance algorithms for CD, particularly during the first year after resection [89].

These findings are consistent with earlier pooled evidence, as a prior systematic review with meta-analysis reported an overall sensitivity of 94% and specificity of 84% (diagnostic accuracy ~90%) for IUS in detecting postoperative recurrence in CD. In subgroup analyses, SICUS demonstrated higher sensitivity (up to 99%) but lower specificity compared with IUS, while a BWT cutoff ≥ 5.5 mm showed high specificity (97.7%) for predicting severe recurrence (Rutgeerts ≥ i3). Overall, IUS demonstrated moderate agreement with endoscopy (κ approximately 0.5–0.8) and appeared to have stronger prognostic value when performed within the first 12 months post-surgery [90].

In line with these findings, the combination of IUS parameters, particularly BWT, with other non-invasive modalities, including FC, emerges as a highly effective non-invasive strategy for early risk stratification in the postoperative setting. This integrated approach significantly enhances diagnostic accuracy, allowing reliable identification of patients at low risk of recurrence and thereby reducing the need for unnecessary colonoscopies. Supporting this concept, a multicenter prospective study conducted across three Italian referral centers, using the Rutgeerts score as the reference standard, identified BWT, mesenteric lymphadenopathy, and FC ≥ 50 μg/g as independent predictors of endoscopic recurrence. Notably, the combined use of BWT ≥ 3 mm and FC ≥ 50 μg/g enabled accurate patient classification and optimized risk stratification [91]. These data are consistent with current recommendations from the ECCO-ESGAR-ESP-IBUS guidelines, which endorse the integration of cross-sectional imaging, such as IUS or MRE, in combination with FC within 3–6 months after ileocecal resection for early detection of recurrence [92]. A structured follow-up remains essential, and ileocolonoscopy is still recommended within 6–12 months postoperatively to confirm endoscopic recurrence and guide therapeutic decisions. Nevertheless, IUS can be used to guide therapeutic decision-making, especially when BWT exceeds 5.5 mm, which may justify therapy start or optimization [93].

IUS is also a valuable tool for supporting the postoperative management of patients with UC who have undergone proctocolectomy with ileal pouch–anal anastomosis (IPAA). In this setting, TPUS has shown promise for diagnosis and monitoring, given its capacity to enable real-time visualization of the pouch, allowing assessment of pouch wall thickness. Recent evidence supports its diagnostic performance, with an AUC of approximately 0.79 for identifying inflammation; a pouch wall thickness < 3 mm is highly sensitive for excluding pouchitis, whereas a thickness ≥4 mm is specific for its diagnosis [94]. Clinically, TPUS is particularly useful when pouchoscopy is not readily available or is contraindicated, and it serves as a complementary tool alongside clinical evaluation and fecal biomarkers. It also plays a relevant role in longitudinal monitoring and in the assessment of pouch-related complications that may be inadequately visualized endoscopically, such as perianal fistulas and pelvic sepsis [95]. Nevertheless, current guidelines emphasize that endoscopy remains the gold standard for the diagnosis and management of pouchitis, particularly in recurrent, refractory, or atypical cases and when CD-like disease of the pouch is suspected. Thus, TPUS should be considered an adjunctive modality, preferably in centers with dedicated expertise [95].

Beyond pouch disorders, TPUS has an established and increasingly recognized role in the evaluation and management of perianal fistulas in CD. It provides high-resolution, real-time imaging of fistulous tracts, internal openings, secondary extensions, and associated abscesses, with good sensitivity and positive predictive value. TPUS is particularly advantageous in the presence of anal canal stenosis or when MRI is unavailable or contraindicated, and it can support both diagnostic classification and therapeutic planning, including guidance for surgical interventions such as seton placement [95,96]. Importantly, TPUS is well suited for serial monitoring, allowing dynamic assessment of treatment response in a patient-friendly and cost-effective manner. Current guidelines acknowledge TPUS as a viable alternative to MRI and endoanal ultrasound in selected settings, particularly for initial evaluation and follow-up, although MRI remains the reference standard for complex or deep pelvic disease [97].

In summary, IUS, including both IUS and TPUS, represents a versatile and accessible imaging approach across the pre- and postoperative spectrum of IBD, especially when integrated with clinical assessment and biomarkers. While endoscopy and MRI remain reference standards in selected scenarios, the complementary use of ultrasound techniques can optimize patient management, reduce the need for invasive procedures, and enhance real-world decision-making [95,96,97].

## 4. Discussion

The management of IBD has progressively evolved from symptom-driven approaches toward objective monitoring strategies aimed at achieving sustained disease control and preventing long-term bowel damage. Within this paradigm shift, IUS has emerged as a valuable tool capable of providing real-time assessment of disease activity while simultaneously addressing many of the practical limitations associated with conventional monitoring modalities. [32,98]. One of the most relevant advantages of IUS is its ability to support a truly dynamic approach to disease management. The growing body of evidence reviewed in this article indicates that sonographic changes frequently occur early after treatment initiation and may provide objective information on treatment effectiveness [14,16,17]. This characteristic makes IUS particularly attractive within precision medicine and T2T strategies, where timely treatment optimization is essential to maximize therapeutic benefit [77,99].

Beyond monitoring inflammatory activity, IUS offers a broader evaluation of disease burden by assessing transmural involvement and disease-related complications. This is particularly relevant in CD, where transmural inflammation, stricturing behavior, and penetrating complications are key determinants of long-term outcomes [14]. At the same time, increasing evidence supports the role of IUS in UC, where ultrasound findings correlate with disease activity and treatment response and may complement endoscopic assessment [65]. The development of TPUS has further expanded the potential applications of ultrasound, particularly in patients with rectal involvement and perianal disease, as well as in the postoperative setting, like in pouch disorder [95].

**Figure 1:**
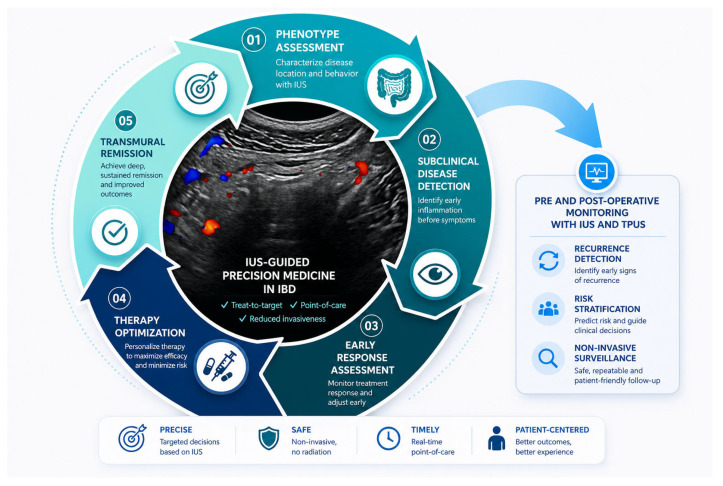
IUS-guided precision medicine in IBD: the central role of IUS in phenotype assessment, subclinical disease detection, early response assessment, therapy optimization, transmural remission assessment and preoperative or postoperative monitoring.

Another important aspect emerging from the available literature is the growing integration of IUS into routine clinical decision-making. Point-of-care assessment, repeated evaluation during follow-up, and integration with biomarkers and therapeutic drug monitoring have the potential to facilitate more personalized treatment strategies. In this context, IUS should not be viewed as a replacement for endoscopy or MRE but rather as a complementary modality capable of providing additional information while reducing the need for invasive or resource-intensive investigations in selected clinical scenarios [36] (Figure 1).

### Limitations and Barriers to Widespread Clinical Implementation of IUS

Despite the growing body of evidence supporting the use of IUS in IBD and its endorsement by international guidelines, several barriers continue to limit its widespread implementation in routine clinical practice. Importantly, these challenges relate less to diagnostic performance and more to structural, educational, and organizational factors.

One of the major limitations of IUS is its operator dependency. Although studies have consistently demonstrated high diagnostic accuracy in experienced hands, achieving adequate proficiency requires dedicated training and a structured learning pathway. In a recent prospective multicenter study conducted across eight tertiary IBD centers, gastroenterologists with extensive abdominal ultrasound experience but limited prior exposure to IUS underwent a standardized training program followed by supervised and independent examinations. While basic sonographic parameters, such as BWT, vascularity, and wall stratification, achieved substantial interobserver agreement early during training, more advanced findings, including strictures, fistulas, and collections, required a longer learning curve. Overall, the study demonstrated that structured training enables acquisition of basic IUS competency after approximately 69–112 examinations, whereas advanced skills require additional experience and ongoing practice [100]. These findings highlight the importance of standardized training programs and certification pathways to ensure reproducibility and facilitate the safe implementation of IUS in routine clinical practice.

Training opportunities also remain unevenly distributed worldwide. While IUS has become an integral component of IBD care in several European centers, its adoption remains limited in many regions, including Asia-Pacific countries, and other healthcare systems where formal training pathways are not yet widely available [101,102]. National surveys consistently identify lack of structured educational programs as one of the main barriers to broader implementation [103,104]. An additional consideration relates to the generalizability of the current evidence base. Most studies evaluating IUS in IBD have been conducted in European tertiary referral centers with dedicated expertise and established ultrasound programs. Consequently, the applicability of these findings to community-based settings, primary care environments, and healthcare systems with limited access to specialized training may be more uncertain. Furthermore, data from Asian populations and other underrepresented regions remain comparatively scarce. Broader international studies involving diverse healthcare settings and patient populations are therefore needed to confirm the reproducibility and real-world applicability of current evidence [102].

Beyond training, important system-level challenges persist. Limited access to dedicated ultrasound equipment, lack of institutional investment, unclear reimbursement policies, and time constraints may hinder the incorporation of IUS into routine outpatient workflows. In many healthcare settings, established diagnostic pathways based on endoscopy and MRE continue to dominate clinical practice, creating resistance to the adoption of newer point-of-care imaging strategies [102].

Another important limitation relates to standardization. Although several ultrasound activity scores have been proposed, there is still no universally accepted scoring system or definition for key sonographic endpoints, including TH. Differences in acquisition techniques, reporting standards, and outcome definitions continue to limit comparability across studies and may partially explain the heterogeneity observed in the literature [102,105].

Technical limitations should also be acknowledged. Visualization of the rectum and proximal jejunum may be suboptimal with transabdominal ultrasound, and image quality can be affected by obesity or excessive bowel gas. However, the limitation of rectum assessment may be overcome with the implementation of TPUS application. Furthermore, although IUS performs well in the assessment of transmural inflammation and disease complications, endoscopy remains superior for the direct evaluation of mucosal lesions and histological assessment, while MRE continues to play a key role in selected patients with complex penetrating disease or deep pelvic involvement [102,105].

Finally, important evidence gaps remain. Although the evidence supporting IUS continues to expand, most available data derive from observational studies and prospective cohorts, while randomized controlled trials evaluating IUS-guided management strategies remain scarce. Further research is needed to standardize sonographic endpoints, validate imaging-based treatment targets, and clarify the role of advanced ultrasound techniques, including CEUS and elastography. Ultimately, the future challenge for IUS is no longer demonstrating diagnostic accuracy but achieving greater standardization, wider dissemination of training, and effective integration into routine clinical care and treat-to-target strategies.

## 5. Conclusions

IUS has emerged as a versatile and increasingly important tool in the management of IBS, offering a non-invasive, repeatable, and patient-friendly approach to disease assessment. By providing real-time information on bowel inflammation, transmural disease involvement, and treatment response, IUS has the potential to complement existing monitoring strategies and support more personalized clinical decision-making across different stages of disease. Although important challenges remain regarding standardization, training, and implementation, the growing body of evidence supporting its clinical utility suggests that IUS will play an increasingly prominent role in future IBD care. Continued efforts to harmonize sonographic definitions, validate clinically meaningful imaging targets, and integrate ultrasound into multidisciplinary monitoring pathways will be essential to fully realize its potential. As these challenges are addressed, IUS is likely to become an integral component of modern, patient-centered IBD management.

## Figures and Tables

**Table 1:** Comparison of intestinal ultrasound (IUS), magnetic resonance enterography (MRE), and endoscopy for disease monitoring in inflammatory bowel disease.

Domain	IUS	MRE	Endoscopy
**Assessment of mucosal** **healing**	Yes (Indirect)	Yes (Indirect)	Gold standard
**Assessment of** **transmural healing**	Yes	Yes	Not possible
**Assessment of strictures**	Yes	Yes	Limited
**Assessment of** **fistulas/abscesses**	Yes	Yes	Limited
**Assessment of mesenteric involvement**	Yes	Yes	Not possible
**Correlation with** **endoscopic activity**	Yes	Yes	Reference standard
**Monitoring treatment** **response**	Yes	Yes	Yes
**Suitability for tight** **disease monitoring**	Excellent	Moderate	Limited
**Point-of-care availability**	Yes	No	No
**Repeatability**	Excellent	Moderate	Limited
**Time required**	10–20 min	30–60 min (+preparation)	20–45 min (+preparation + recovery time)
**Patient burden**	Low	Moderate	High
**Need for patient** **preparation**	Minimal	Moderate–high (fasting, oral contrast, antiperistaltic agents)	High (bowel cleansing ± sedation)
**Need for sedation**	No	No	Yes
**Radiation exposure**	None	None	None
**Ability to obtain** **histology**	No	No	Yes
**Cost**	Low	High	Moderate–high
**Accessibility**	High, especially in referral centers	Variable	Variable
**Safety profile**	Excellent; non-invasive, no radiation, no contrast agents required	No radiation but requires oral contrast administration	Invasive procedure with established risks related to bowel preparation, sedation, bleeding, and perforation
**Evaluation of post** **operative recurrence**	Yes	Yes	Current reference standard
**Role in treat-to-target** **strategies**	Emerging central tool for serial monitoring and transmural assessment	Complementary reference imaging modality for transmural and extramural disease evaluation	Current reference standard for mucosal healing and therapeutic targets
**Major strengths**	Real-time assessment, non-invasive, repeatable, inexpensive, patient-friendly	Comprehensive transmural and extramural assessment, high diagnostic accuracy	Direct visualization of mucosa, histological sampling possible
**Major limitations**	Operator dependence, limited visualization of deep pelvic and proximal small bowel segments	Cost, availability, longer examination time, need for preparation	Invasive, patient burden, need for bowel preparation, evaluates mucosa only

**Table 2:** Formulae and sonographic parameters included in the most commonly used intestinal ultrasound activity scores for inflammatory bowel disease.

Score	Disease	Formula	Parameters
**IBUS-SAS**	CD, UC	4 × BWT + 15 × i-fat + 7 × CDS + 4 × BWS	BWT (continuous, mm); CDS: 0 = absent, 1 = short signals, 2 = long signals inside bowel wall, 3 = long signals inside and outside bowel wall; BWS: 0 = normal, 1 = uncertain, 2 = focal loss (≤3 cm), 3 = extensive loss (>3 cm); i-fat: 0 = absent, 1 = uncertain, 2 = present
**BUSS**	CD	0.75 × BWT + 1.65 × CDS	BWT (continuous, mm); CDS: 0 = absent, 1 = present
**SUS-CD**	CD	BWT score + CDS score	BWT: 0 = ≤3 mm, 1 = 3–4.9 mm, 2 = 5–7.9 mm, 3 = ≥8 mm; CDS: 0 = no vessels/cm^2^, 1 = 1–2 vessels/cm^2^, 2 = ≥3 vessels/cm^2^
**MUC**	UC	1.4 × BWT + 2 × CDS	BWT (continuous, mm); CDS: 0 = absent, 1 = present

**Abbreviations:** BWT, bowel wall thickness; CDS, color Doppler signal; BWS, bowel wall stratification; i-fat, inflammatory fat; IBUS-SAS, International Bowel Ultrasound Segmental Activity Score; BUSS, Bowel Ultrasound Score; SUS-CD, Simple Ultrasound Score for Crohn’s Disease; MUC, Milan Ultrasound Criteria.

## Data Availability

No new data were created or analyzed in this study. Data sharing is not applicable to this article.

## Abbreviations

The following abbreviations are used in this manuscript:

ASUC	Acute Severe Ulcerative Colitis
AUC	Area Under the Curve
AUROC	Area Under the Receiver Operating Characteristic Curve
BUSS	Bowel Ultrasound Score
BWT	Bowel Wall Thickness
BWS	Bowel Wall Stratification
CD	Crohn’s Disease
CDS	Color Doppler Signal
CEUS	Contrast-Enhanced Ultrasound
CI	Confidence Interval
CRP	C-Reactive Protein
CT	Computed Tomography
CTE	Computed Tomography Enterography
ECCO	European Crohn’s and Colitis Organisation
ESGAR	European Society of Gastrointestinal and Abdominal Radiology
ESP	European Society of Pathology
FC	Fecal Calprotectin
IBD	Inflammatory Bowel Disease
IBUS	International Bowel Ultrasound Group
IBUS-SAS	International Bowel Ultrasound Group Segmental Activity Score
ICC	Intraclass Correlation Coefficient
IPAA	Ileal Pouch–Anal Anastomosis
ITL	Infliximab Trough Level
IUS	Intestinal Ultrasound
IVCS	Intravenous Corticosteroids
MH	Mucosal Healing
MRI	Magnetic Resonance Imaging
MRE	Magnetic Resonance Enterography
MUC	Milan Ultrasound Criteria
OR	Odds Ratio
POC	Point-of-Care
SICUS	Small Intestine Contrast Ultrasonography
SES-CD	Simple Endoscopic Score for Crohn’s Disease
STRIDE	Selecting Therapeutic Targets in Inflammatory Bowel Disease
SUS-CD	Simple Ultrasound Activity Score for Crohn’s Disease
SWE	Shear Wave Elastography
T2T	Treat-to-Target
TDM	Therapeutic Drug Monitoring
TH	Transmural Healing
TNF	Tumor Necrosis Factor
TPUS	Transperineal Ultrasound
UC	Ulcerative Colitis
WLS	Wall Layer Stratification
